# The 2017 Keystone Symposium on HIV Vaccines

**DOI:** 10.1080/21645515.2017.1353852

**Published:** 2017-08-08

**Authors:** Christopher A. Cottrell, Andrew B. Ward

**Affiliations:** Department of Integrative Structural and Computational Biology, Scripps Center for HIV/AIDS Vaccine Immunology and Immunogen Discovery (CHAVI-ID), International AIDS Vaccine Initiative (IAVI) Neutralizing Antibody Center, and Collaboration for AIDS Vaccine Discovery (CAVD), The Scripps Research Institute, La Jolla, CA, USA

**Keywords:** adjuvant, antibody, bnAb, germline, HIV vaccine

## Introduction

The Keystone Symposium on HIV Vaccines (meeting #C9) brought together more than 300 researchers from around the global in Steamboat Springs, Colorado from March 26^th^ to March 30^th^ 2017. The event was organized by Drs. Andrew Ward (The Scripps Research Institute [TSRI]), Penny Moore (University of the Witwatersrand), and Robin Shattock (Imperial College London), to discuss the latest results from human clinical studies, along with the cutting-edge basic science behind such trials to highlight approaches that may lead to an effective HIV vaccine. The specific aims of the meeting were: (1) To present and discuss the most current concepts and findings relevant to the design of an HIV vaccine, including studies of infected cohorts and animal models. (2) To assess and debate emerging data from animal and human clinical trials testing novel concepts and products with particular focus on evaluating the underlying basic science that underpin these trials. (3) To enable and enhance existing and new collaborations between basic and clinical scientists within the broad field of HIV vaccinology and from outside of the field, by bringing together scientists with diverse expertise and backgrounds in a focused environment conducive to robust discussion. (4) To provide trainees and new investigators an opportunity to interact with their peers from across the world, present their data and network with established investigators. Inclusion of young and emerging investigators in all aspects of the scientific program created opportunities for junior scientists to present their work. Recurrent topics during the meeting included: isolation and characterization of broadly neutralizing antibodies (bnAbs) from HIV-1 infected patients; HIV immunogen design and pre-clinical trials; clinical trials; novel adjuvants; and technology development.

### Isolation and characterization of bnAbs

BnAbs have been shown to be protective against infection in animal models, and eliciting such antibodies is the aspirational target of many HIV vaccine programs.^1,2^ Nearly all bnAbs that have been discovered thus far were isolated from individuals with chronic HIV infection and demonstrate that the human immune system is capable of generating a broad and potent anti-HIV humoral response.^3^ However, bnAbs alone do not provide information on the maturation process that led to the development of breadth and potency. Longitudinal studies, particularly studies that enroll subjects before infection, provide an opportunity to examine the complex interplay between viral evolution and the developing antibody lineages.^4-6^ Elise Landais (International AIDS Vaccine Initiative [IAVI)] and Sasha Murrell (TSRI) presented data describing several new bnAb lineages isolated from patients within the IAVI Proctcol C cohort that target the N332 supersite, the CD4bs, or the V2-apex. Evan Cale (National Institutes of Health-Vaccine Research Center [NIH-VRC]) presented data on a new quaternary specific, 35O22-like bnAb (VRC44.01), isolated from the same patient that also developed the V2-apex targeting bnAb VRC38.^7^ Nicole Doria-Rose (NIH-VRC) presented the first lineage analysis of a membrane proximal external region (MPER) targeting bnAb.

In addition to describing newly isolated bnAbs, several investigators presented data detailing factors that contribute to development of bnAbs in some individuals. Julie Overbaugh (Fred Hutchinson Cancer Research Center [FHCRC]) presented data on the development of bnAbs in the context of mother-to-child HIV transmission, demonstrating that viruses transmitted from the mother were most often escape variants that were not neutralized by temporally matched maternal serum. Infants who went on to develop broadly neutralizing serum did so at a faster rate with more polyclonality compared with adults. Monoclonal bnAbs isolated from these infants had undergone less somatic hypermutation when compared with bnAbs isolated from adults.^8^ One possible explanation for the rapid, polyclonal development of breadth in neonates is the evolutionary pressure provided by passively transferred maternal IgG. In the mother-to-child transmission context, the transmitted founder virus in the infant is simultaneously under selective pressure from both maternal antibodies and the de novo immune response of the infant. While the specificity of the maternal antibodies remains static, the amount of maternal IgG in the infant declines over time. Alexandra Trkola (University of Zurich) described a large study from a Swiss HIV cohort that examined correlates of bnAb development that found viral load, length of untreated infection, and viral diversity drove bnAb development independently.^9^ Penny Moore (University of the Witwatersrand) described data from the CAPRISA cohort showing patients that developed bnAbs did so in the first 3–4 y of infection and that breadth was frequently linked to high viral load, high viral diversity and rapid escape from initial neutralizing antibodies. Additionally, Dr. Moore presented data showing bnAb lineages persisted in patients even after viral escape, suggesting the continual presence of Env antigens capable of binding and stimulating the bnAb lineages.

### HIV immunogen design and pre-clinical trials

Barton Haynes (Duke University Medical Center) presented a series of studies describing the use of CH505 lineage based immunogens in CH103 germline knock-in mice, rabbits, and rhesus macaques and their planned eventual use in human clinical trials. Todd Bradley (Duke University Medical Center) gave a short talk on the effect of co-administering PD-1 and CTLA4 checkpoint inhibitors with CH505 gp120 immunogens. Peter Kwong (NIH-VRC) showed results from immunizations studies using keyhole limpet hemocyanin (KLH)-HIV fusion peptide immunogens in mice and rhesus macaques, demonstrating elicitation of antibodies targeting the fusion peptide with modest neutralization capability. Jon Steichen (TSRI) described a sequential series of immunogens used to target and expand B-cells in PGT121 germline knock-in mice.^10,11^ There were several additional talks and posters describing the design of germline targeting immunogens. Rogier Sanders and Maximilliano Medina-Ramirez (University of Amsterdam) described their work modifying the BG505 SOSIP immunogen to simultaneously target the germline precursors of CD4 binding site and V1/V2 apex bnAbs. Marie Pancera (FHCRC) described a method for identifying and quantifying genuine bnAb germline precursor B-cells in naïve subjects using bnAb anti-idiotypic antibodies as cell sorting probes. Colin Havenar-Daughton (La Jolla Institute of Allergy and Immunology) and Matthias Pauthner (TSRI) presented results from a large Scripps CHAVI-ID rhesus macaque study showing that subcutaneous immunization of rhesus macaques at 0, 8, and 24 weeks with BG505 SOSIP consistently elicited higher titers of autologous neutralizing antibodies when compared with intramuscular immunization or immunizations with a shorter interval between the prime and first booster.^12^

### Clinical trials

Nina Russell (Bill and Melinda Gates Foundation) gave an outstanding keynote address outlining the historical arc of HIV vaccine efficacy trials and providing a preview of ongoing and planned clinical trials. Mark Feinberg (IAVI) told the inspiring story of how a global community, consisting of both public and private entities, came together to rapidly create an Ebola vaccine. His narrative offered insights into the process of vaccine development as well as cautionary notes about the importance of seeing product development through to the end. Till Schoofs (Rockefeller University) presented the results from a passive immunization study that administered the N332 targeting bnAb 10-1074 to HIV-1 infected individuals. Peak suppression of viral load occurred 7 d after administration, after which the viral load gradually returned to baseline due to viral escape from 10–1074 and clearance of the antibody.^13^ Juliana McElrath (FHCRC) presented an update on the results of the HVTN100 study and its use as a go/no-go criteria for launching the Phase 3 efficacy trial HVTN702. The pre-specified HVTN702 “go” criteria were: (1) prevalence of IgG binding antibodies to at least 2 of 3 gp120 vaccine antigens in ≥ 75% of participants; (2) non-inferior IgG binding antibody magnitude to gp120 vaccine antigens compared with the results from the RV144 trial; (3) non-inferior response rate of Env-specific CD4+ T-cells response compared with the results from the RV144 trial; and (4) prevalence of IgG binding antibodies to at least 1 clade C V1/V2 antigen in ≥ 56% of participants. Results from the HVTN100 trial showed all 4 “go” criteria were met leading to the launch of the HVTN702 trial in October of 2016.

### Adjuvants and technology development

Carl Alving, (Walter Reed Army Institute of Research) and Mark Orr (Infectious Disease Research Institute [IDRI]) described the Army's liposomal formulation and IDRI's GLA-SE adjuvant systems, respectively.^14,15^ The Army's liposomal formulation (ALF) consisted of small unilamellar vesicle liposomes containing neutral and anionic saturated phospholipids, cholesterol, and the toll-like receptor-4 (TLR-4) agonist monophosphoryl lipid A. The ALF adjuvant was formulated with or without the saponin QS-21 (ALFQ) and administered to mice with a CN54gp140 HIV Env trimer immunogen. The ALFQ version produced a more balanced Th1/Th2 immune response compared the ALF adjuvant without QS-21 as measured by antibody subtype production and cytokine secretion.^15,16^ The GLA-SE adjuvant system consisted of glycopyranosyl lipid adjuvant (GLA), a synthetic monophosphoryl lipid A analog, formulated in a stable squalene-in-water emulsion (SE). The GLA-SE adjuvant system was evaluated in mice using a *Mycobacterium tuberculosis* antigen. Immunization with GLA-SE produced more antigen specific B-cells, higher antibody binding titers, and more CD4+ T-follicular helper and CD4+ Th1 cells compared with alum, GLA without SE, or SE alone.^14^ Darrell Irvine (Massachusetts Institute of Technology [MIT]) described a method for manipulating the germinal center reaction using an escalating dose of antigen and described novel technologies to facilitate such manipulation, including microneedle patches and small molecule regulated RNA expression.^17^ Alejandro Balazs (MIT) described a novel vector system for antibody gene delivery and immunoprophylaxis using Cre-LoxP as a built-in off switch of gene expression. Neil King (University of Washington) described a series of self-assembling nanoparticles that could be decorated with HIV trimer immunogens to present B-cells with a multitude geometric valences including tetrahedral, octahedral or icosahedral antigen display.^18,19^ Robin Shattock (Imperial College London) described small human experimental medicine trials using HIV Env encoding DNA vaccines and emphasized the rapid and iterative nature with which DNA immunization could be conducted in humans compared with protein based immunizations. Gunilla Karlsson Hedestam (Karolinska Institutet) presented a method of determining individual VH germline repertoires using next generation sequencing. This method (IgDiscover) was used to elucidate the germline B-cell repertoires of rhesus macaques and healthy human volunteers demonstrating a higher level of variation among individuals and identifying multiple novel VH alleles for both species.^20^ Brandon DeKosky (NIH) presented a method for high-throughput paired immunoglobulin heavy and light chain sequencing of single sorted memory B-cells that used a flow based system for isolating individual B-cells in single droplet emulsions.^21^ Stephen Kent (University of Melbourne) developed a novel Fc-receptor dimer assay for measuring Fc mediated effector functions of anti-HIV antibodies derived from natural infection or elicited by vaccination.^22^ The Fc-receptor dimer assay could be conducted in a robust and reproducible manner with results that were highly correlated with results from several different assay types that measured antibody-dependent cell-mediated cytotoxicity (ADCC).^22^ The Global HIV Vaccine Enterprise held a workshop during the symposia entitled, “Knowns and Unknowns of Assaying ADCC against HIV” that discussed the various ADCC assays used to assess immune responses induced by HIV vaccine candidates in clinical and pre-clinical trials. The overall conclusion of the workshop was that no single ADCC assay fully recapitulated the *in vivo* ADCC process and that efforts should be made to ensure ADCC assays utilize autologous reagents (antibodies, effector cells, target cells, and viral strain) to the maximum extent possible.

### Conclusion

Above is a small sampling of the 55 informative presentations given during the meeting. In addition to the formal presentations, multiple poster sessions and networking events facilitated discussion of new and unpublished data, enabling the formation of new collaborations and the sharing of resources. Overall, the meeting successfully accomplished its specific aims and illuminated future directions in the HIV vaccine field including:

• Initiation of the first in human trial with native HIV Env trimers
• Validation of pre-clinical animal models systems
• Analysis of the on-going HVTN702 Phase 3 efficacy trial
• Continual advancement of structure-based immunogen design
• Continual isolation and characterization of novel bnAbs and bnAb lineages
